# Early adverse events and patient-reported outcomes after salvage radiotherapy following prostatectomy according to fractionation schedule: Interim analysis of the phase III URONCOR 06–24 trial

**DOI:** 10.1016/j.ctro.2026.101276

**Published:** 2026-08-30

**Authors:** Felipe Couñago, Carmen González, Alfonso Gómez-Iturriaga, Ana Álvarez, Marina Santos, Ana Boladeras, Nagore García, Sandra Fernández, Sandra Guardado, Abrahams Ocanto, Daniela Gonsalves, Luis Glaría, Ana Castaño, Patricia Martín, Laura Montezuma, Arantxa Mera, Joel Mases, Gerard Meca, Víctor Duque-Santana, Jesús Olivera, Sara Pérez, Ivan Henriquez, Bárbara Malavé, Aurora Rodríguez, Rafael García, David Büchser, Josep Garre, Manuel Altabas, Jeannette Valero, Raquel García-Gómez, Miguel Berenguer, Ángel Calvo, Rafael Ordoñez, Patricia Willisch, Aldara Candal, Neus Aymar, Esther Mayrata, Fernando López-Campos

**Affiliations:** a Department of Radiation Oncology, Vithas La Milagrosa University Hospital, Madrid 28010, Spain; b Department of Radiation Oncology, San Francisco de Asís University Hospital, Madrid 28002, Spain; c Department of Medicine, Faculty of Medicine, Health and Sport Sciences, Universidad Europea de Madrid, Spain; d National Director of GenesisCare, Spain.; e Department of Radiation Oncology, Gregorio Marañón University General Hospital, Madrid 28007, Spain; f Department of Radiation Oncology, Cruces University Hospital, Barakaldo 48903, Spain.; g Biobizkaia Health research institute, Barakaldo 48903, Spain; h Universidad del País Vasco (UPV/EHU), Spain; i Department of Radiation Oncology, Catalan Institute of Oncology Hospitalet, Barcelona 08908, Spain; j Department of Radiation Oncology, 12 de Octubre University Hospital, Madrid 28041, Spain; k Department of Radiation Oncology, La Paz University Hospital, Madrid 28046, Spain; l Department of Radiation Oncology, Ramón y Cajal University Hospital, Madrid 28034, Spain; m Department of Radiation Oncology, Santa Creu i Sant Pau Hospital, Barcelona 08025, Spain; n Department of Radiation Oncology, Hospital Clinic Barcelona, Barcelona 08036, Spain; o Department of Radiation Oncology, Quirónsalud University Hospital, Madrid 28003, Spain; p Department of Radiation Oncology, Fundación Jiménez Díaz University Hospital, Madrid 28040, Spain; q Department of Radiation Oncology, San Joan Reus University Hospital, Tarragona 43204, Spain; r Institut de Recerca Biomèdica Catalunya Sud (IRB-CS), Spain; s Department of Radiation Oncology, Viamed Tarragona Hospital, Tarragona 43006, Spain; t Department of Radiation Oncology, Ruber International Hospital, Madrid 28034, Spain; u Department of Radiation Oncology, Vall d'Hebron University Hospital, Barcelona 08035, Spain; v Department of Radiation Oncology, HM Sanchinarro University Hospital, Madrid 28050, Spain; w Department of Radiation Oncology, La Fe University and Polytechnic Hospital, Valencia 46026, Spain; x Department of Radiation Oncology, Vithas Consuelo Hospital, GenesisCare, Valencia 46007, Spain; y Department of Radiation Oncology, Virgen de la Victoria University Hospital, Málaga 29010, Spain; z Department of Radiation Oncology, Meixoeiro Hospital, Vigo 36214, Spain; aa Department of Radiation Oncology, Son Espases University Hospital, Palma de Mallorca 07010, Spain

**Keywords:** Prostate cancer, Salvage radiotherapy, Moderate hypofractionation, Side effects, Patient-reported outcomes

## Abstract

**Background and purpose:**

Moderate hypofractionation is increasingly used in salvage radiotherapy (SRT) after radical prostatectomy, although prospective data in the postoperative setting remain limited. We evaluated early adverse events and patient-reported outcomes (PROs) according to fractionation schedule within a prospective multicenter phase III trial.

**Materials and methods:**

This prespecified interim analysis included the first 250 patients enrolled in the URONCOR 06–24 randomized trial (NCT05781217). SRT was delivered using moderate hypofractionation (*n* = 175), most commonly 62.5 Gy in 25 fractions (80.5%), followed by 65.25 Gy in 29 fractions (7.5%), 60 Gy in 24 fractions (4.6%), and 52.5 Gy in 20 fractions (2.9%), corresponding to a biologically equivalent dose (EQ.D2) of approximately 65–71 Gy (α/β = 1.5), or conventional fractionation (*n* = 75; 66–70 Gy in 33–35 fractions). Adverse events were assessed using CTCAE v5.0. PROs were evaluated using EPIC-26 and EORTC QLQ-C30 at baseline, 3, and 6 months.

**Results:**

With a median follow-up of 12.7 months, grade ≥ 2 genitourinary (GU) adverse events occurred in 21.7% of patients treated with hypofractionation versus 17.3% with conventional fractionation (*p* = 0.431). Grade ≥ 2 gastrointestinal (GI) adverse events occurred in 10.8% and 4.0%, respectively (*p* = 0.079). Pelvic nodal irradiation was more frequent in the hypofractionation group (39.4% vs 13.3%, *p* = 0.001). On multivariable analysis, age was associated with increased risk of GU ≥2 adverse events, while pelvic nodal irradiation was associated with increased risk of GI ≥2 adverse events. No clinically meaningful differences were observed in PROs.

**Conclusion:**

Early adverse events after SRT were low and comparable between fractionation schedules, and moderate hypofractionation showed a similar safety profile despite more frequent pelvic irradiation. However, given the non-randomized nature of the fractionation schedules, longer follow-up and robust adjustment for treatment-selection biases are needed before drawing definitive conclusions regarding comparative safety.

## Highlights

• Prospective multicenter interim analysis from the phase III URONCOR 06–24 trial.
• Moderate hypofractionated salvage radiotherapy showed low early GU and GI side effects.
• Side effects and patient-reported outcomes were comparable to conventional fractionation.
• Similar safety was observed despite more frequent pelvic nodal irradiation in the hypofractionation.

## 1 Introduction

Salvage radiotherapy (SRT) to the prostate bed is the standard treatment for patients with biochemical recurrence after radical prostatectomy and represents the only potentially curative option in this setting. Several studies have demonstrated that early SRT improves biochemical control and long-term oncological outcomes when delivered at low prostate-specific antigen (PSA) levels [1], [2], [3]. Consequently, SRT has become a cornerstone of postoperative management for prostate cancer.

Over the past decade, moderate hypofractionation has emerged as an attractive strategy in prostate cancer radiotherapy. This approach is supported by the radiobiological hypothesis of a low α/β ratio for prostate cancer [4] and by multiple randomized trials demonstrating non-inferior tumor control and comparable side effects compared with conventional fractionation in the primary treatment setting [5], [6], [7]. As a result, moderate hypofractionation has been widely adopted for definitive radiotherapy and is increasingly being implemented in the postoperative setting, including salvage treatment after prostatectomy.

Historically, however, most randomized trials evaluating postoperative radiotherapy have used conventional fractionation schedules, typically delivering 64–72 Gy in daily fractions of 1.8–2 Gy to the prostate bed [8], [9], [10]. Consequently, prospective comparative evidence supporting hypofractionated schedules in the post-prostatectomy setting has been limited.

More recently, randomized data have begun to address this evidence gap. The phase III NRG-GU003 trial compared hypofractionated post-prostatectomy radiotherapy (62.5 Gy in 25 fractions) with conventional fractionation (66.6 Gy in 37 fractions) and demonstrated non-inferiority in patient-reported genitourinary (GU) and gastrointestinal (GI) side effects at two years, although transiently higher GI symptoms were observed at the end of treatment in the hypofractionation arm [11]. Similarly, a randomized phase III trial comparing 52.5 Gy in 20 fractions with conventional postoperative radiotherapy also reported comparable adverse events outcomes between fractionation schedules [12]. Additional prospective studies have further supported the feasibility and safety of moderately hypofractionated postoperative radiotherapy, reporting acceptable adverse events profiles and encouraging early oncological outcomes [13], [14].

In practice, SRT is frequently delivered in combination with systemic therapy and pelvic volumes. Randomized trials such as GETUG-AFU 16 and RTOG 9601 demonstrated that the addition of androgen deprivation therapy (ADT) improves oncological outcomes in selected patients receiving SRT [15], [16]. Furthermore, elective pelvic nodal irradiation is increasingly considered for patients at higher risk of nodal involvement, particularly in the era of advanced molecular imaging. These treatment intensification strategies may influence treatment-related side effects and highlight the importance of evaluating the safety of hypofractionated regimens in contemporary treatment scenarios.

In this context, prospective data evaluating both physician-reported adverse events and patient-reported outcomes (PROs) are essential to guide clinical decision-making, particularly in the postoperative setting where urinary and bowel symptoms may significantly affect quality of life.

URONCOR 06–24 is a prospective, multicenter, randomized phase III trial evaluating the optimal duration of ADT combined with salvage radiotherapy in patients with intermediate- and high-risk biochemical recurrence after prostatectomy. The study design and early characteristics of the trial cohort have been previously reported [[17], [18]]. Within this contemporary prospective cohort, different SRT fractionation schedules are used according to institutional practice, providing an opportunity to evaluate treatment-related side effects and patient-reported outcomes associated with different fractionation approaches.

The aim of the present analysis was to evaluate early genitourinary and gastrointestinal adverse events, as well as patient-reported outcomes, according to fractionation schedule in the first 250 patients enrolled in the URONCOR 06–24 trial. These findings may help inform the ongoing adoption of hypofractionated salvage radiotherapy in contemporary clinical practice.

## 2 Methods

### 2.1 Study design and objectives

URONCOR 06–24 (NCT05781217) is an ongoing prospective, multicenter, open-label, randomized phase III trial designed to evaluate the optimal duration of androgen deprivation therapy (ADT) in combination with salvage radiotherapy (SRT) in patients with biochemical recurrence (BCR) following radical prostatectomy. Patients are randomized in a 1:1 ratio to receive either short-term (6 months) or long-term (24 months) ADT combined with SRT. Both treatment strategies reflect current standards of care and contemporary clinical practice. The primary endpoint is metastasis-free survival (MFS), defined as the time from ADT initiation (first administration of an LHRH analogue) to the development of distant metastatic disease (M1a–c), assessed using conventional imaging (computed tomography and bone scan) according to RECIST 1.1 criteria. The use of molecular imaging (PSMA or choline PET/CT) was permitted at the investigator's discretion according to institutional protocols [[17], [19]]. Secondary endpoints include biochemical relapse-free survival, pelvic progression-free survival, time to systemic therapy, time to castration resistance, cancer-specific survival, overall survival, treatment-related adverse events, and patient-reported outcomes (PROs).

The planned sample size is 534 patients across 18 centers in Spain. Recruitment began in March 2023 and is expected to continue for approximately 4 years, with a minimum follow-up of 5 years for the primary endpoint analysis [[17], [19]]. This analysis represents a prespecified interim analysis of the first 250 randomized patients, focusing on early side effects and PROs.

### 2.2 Study population

Eligible patients had histologically confirmed prostate adenocarcinoma treated with radical prostatectomy and subsequent biochemical recurrence, defined as a prostate-specific antigen (PSA) level ≥ 0.2 ng/mL with at least one confirmatory rising value obtained ≥2 weeks later. Patients were required to have no evidence of macroscopic local recurrence or distant metastatic disease on conventional imaging and to have adequate performance status. The study was approved by the institutional review boards of all participating centers and conducted in accordance with the Declaration of Helsinki. All patients provided written informed consent.

## 3 Radiotherapy

### 3.1 Treatment planning and target delineation

Radiotherapy was delivered using modern external beam techniques following CT-based simulation. Clinical target volumes (CTV) were defined according to internationally recognized post-prostatectomy contouring guidelines (ESTRO-ACROP, RTOG, and EORTC) [[20], [21], [22], [23]]. A planning target volume (PTV) margin of 5–10 mm was applied according to institutional protocols. Elective pelvic nodal irradiation was permitted at the investigator's discretion, particularly in patients with high-risk features or without prior pelvic lymph node dissection. No protocol-mandated criteria were specified, reflecting real-world practice.

### 3.2 Dose and fractionation

Radiotherapy schedules were selected according to institutional protocols, aiming to deliver a biologically equivalent dose in 2 Gy fractions (EQ.D2) of approximately 65–71 Gy (α/β = 1.5).

• Conventional fractionation: 66–70 Gy in 33–35 fractions
• Moderate hypofractionation: 20–29 fractions

The most commonly used hypofractionated regimens included 62.5 Gy in 25 fractions, 65.25 Gy in 29 fractions, 60 Gy in 24 fractions, and 52.5 Gy in 20 fractions, all within the predefined EQ.D2 range. The use of different fractionation schedules reflects contemporary variability in clinical practice across participating centers.

### 3.3 Treatment delivery

Radiotherapy was delivered using intensity-modulated radiotherapy (IMRT), volumetric modulated arc therapy (VMAT), or tomotherapy. Daily image guidance (IGRT) with cone-beam CT (CBCT) or equivalent was mandatory. Dose constraints for organs at risk were applied according to institutional protocols and international recommendations.

### 3.4 Androgen deprivation therapy

ADT was administered according to randomization (6 vs 24 months) and consisted of LHRH analogues (triptorelin, goserelin, or leuprolide). To prevent testosterone flare, bicalutamide (50 mg daily) was administered for 30 days at treatment initiation and subsequently discontinued. ADT was initiated prior to radiotherapy.

### 3.5 Adverse events and patient-reported outcomes

Treatment-related adverse events were prospectively assessed using the Common Terminology Criteria for Adverse Events (CTCAE), version 5.0. Early events were defined as adverse events occurring during radiotherapy and within the first 6 months after treatment initiation. Clinically relevant side effects were defined as grade ≥ 2 events, and grade ≥ 3 events were reported separately. Patient-reported outcomes (PROs) were assessed using the Expanded Prostate Cancer Index Composite-26 (EPIC-26) and the European Organization for Research and Treatment of Cancer Quality of Life Questionnaire Core-30 (EORTC QLQ-C30). Assessments were performed at baseline and at 3 and 6 months after treatment initiation.

### 3.6 Statistical analysis

Descriptive statistics were used to summarize baseline characteristics, treatment parameters, and outcomes. The primary objective of this interim analysis was to compare rates of grade ≥ 2 genitourinary (GU) and gastrointestinal (GI) adverse events according to radiotherapy fractionation schedule (moderate hypofractionation vs conventional fractionation). Categorical variables were compared using the Fisher exact test. Patient-reported outcomes were analyzed longitudinally across predefined time points. Clinically meaningful differences were interpreted according to established thresholds for the EPIC and EORTC QLQ-C30 instruments. Univariate and multivariate logistic regression analyses were performed to identify clinical and treatment-related predictors of grade ≥ 2 GU and GI adverse events. Variables with *p* < 0.10 in univariate analysis were included in multivariate models. Odds ratios (ORs) with 95% confidence intervals (CIs) were reported. A two-sided *p*-value <0.05 was considered statistically significant.

## 4 Results

### 4.1 Patient characteristics

**Fig. 1:**
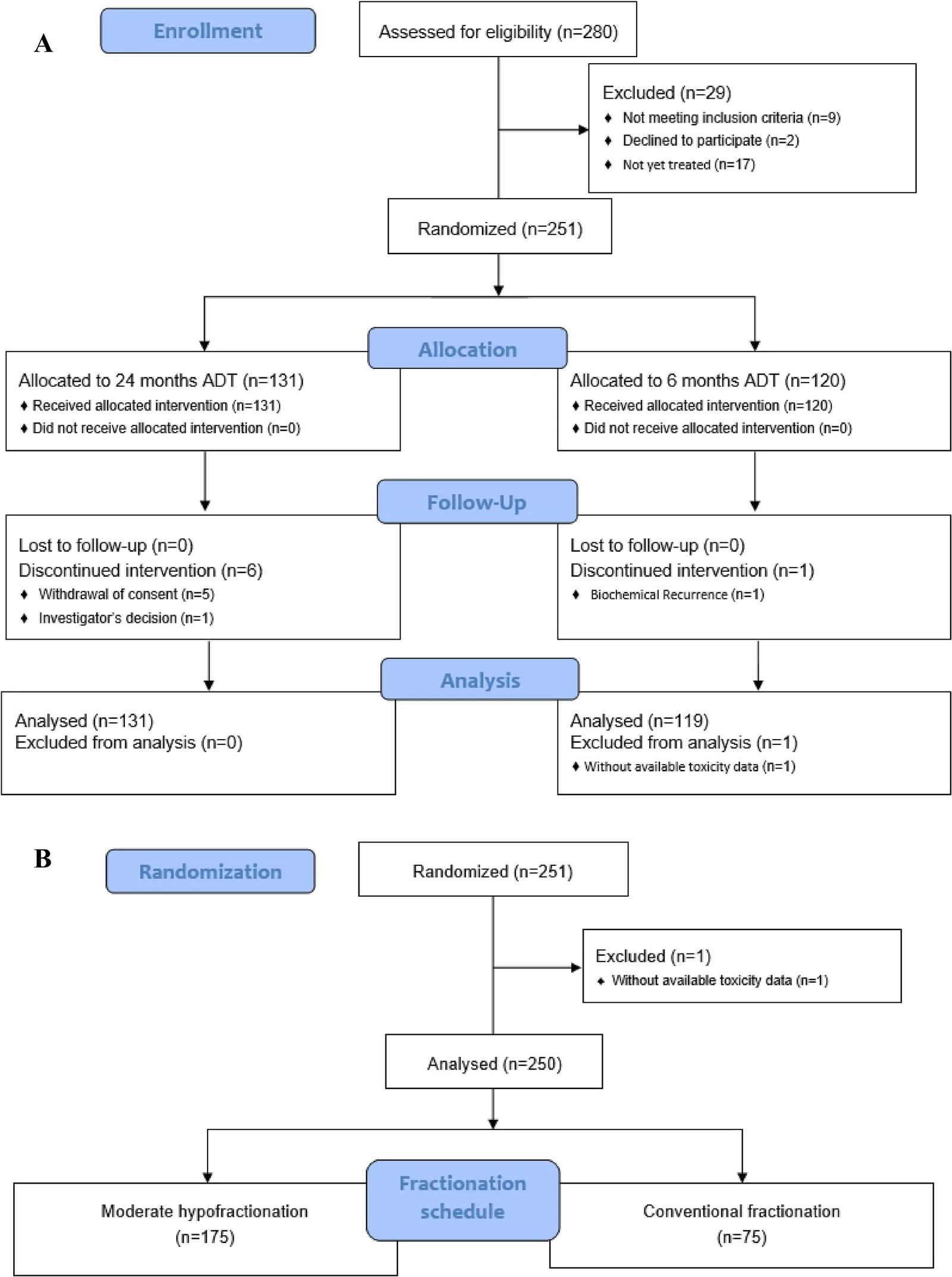
A. Flow-chart showing inclusion, randomization, and participation throughout the study: CONSORT Flow Diagram. B. Flow-chart showing fractionation schedule.

A total of 280 patients were enrolled between March 2023 and February 2026. Of these, 251 patients were randomized in the URONCOR 06–24 trial: 131 to 24 months and 120 to 6 months of androgen deprivation therapy (ADT). The present analysis included the first 250 randomized patients with available adverse events data (Fig. 1A). High-risk disease accounted for 70.5% of the cohort (*n* = 177). The median age was 68 years (IQR 63.5–72.5), the median PSA at study inclusion was 0.34 ng/mL (IQR 0.27–0.49), and the median time from radical prostatectomy to biochemical recurrence (BCR) was 18 months. Molecular imaging with PET/CT was performed in 68.9% of patients (*n* = 173), predominantly using PSMA-based tracers (*n* = 171). Baseline patient and disease characteristics are summarized in Table 1.

**Table 1:** Baseline patient and disease characteristics.

	Total (*N* = 250)	Conventional fractionation (*n* = 75)	Hypofractionation (*n* = 175)	p
Age, median (IQR), years	68.7 (63.7–72.7)	68.7 (63.9–72.2)	68.9 (63.4–73.3)	0.903
ECOG = 1	12 (4.8%)	5 (6.7%)	7 (4.0%)	0.366
Comorbidities				
Hypertension	127 (50.8%)	37 (49.3%)	90 (51.4%)	0.761
Diabetes	33 (13.2%)	13 (17.3%)	20 (11.4%)	0.206
Heart disease	35 (14.0%)	6 (8.0%)	29 (16.6%)	0.073
Kidney disease	8 (3.2%)	1 (1.3%)	7 (4.0%)	0.272
Peripheral artery disease	8 (3.2%)	0 (0.0%)	8 (4.6%)	0.208
Lung disease	17 (6.8%)	6 (8.0%)	11 (6.3%)	0.622
Liver disease	1 (0.4%)	0 (0.0%)	1 (0.6%)	0.535
Cerebrovascular disease	5 (2.0%)	2 (2.7%)	3 (1.7%)	0.622
Other cancer	20 (8.0%)	4 (5.3%)	16 (9.1%)	0.309
PSA at diagnosis, median (IQR), ng/mL	7.0 (5.0–11.0)	7.1 (5.0–12.9)	7.0 (5.0–10.0)	0.312
Gleason score				0.627
≤6	44 (17.7%)	12 (16.2%)	32 (18.4%)	
3 + 4	77 (31.0%)	25 (33.8%)	52 (29.9%)	
4 + 3	59 (23.8%)	20 (27.0%)	39 (22.4%)	
≥8	48 (19.4%)	17 (23.0%)	51 (29.3%)	
Pathologic T stage				0.822
pT2	127 (51.2%)	36 (48.6%)	91 (52.3%)	
pT3a	79 (31.9%)	24 (32.4%)	55 (31.6%)	
pT3b	42 (16.9%)	14 (18.9%)	28 (16.1%)	
Positive margins	155 (66.8%)	43 (62.3%)	112 (68.7%)	0.345
Lymph node involvement				0.806
pN0	137 (55.2%)	40 (54.1%)	97 (55.7%)	
pNx	111 (44.8%)	34 (45.9%)	77 (44.3%)	
NCCN Risk Group at diagnosis				0.750
Low / Favorable Intermediate	97 (40.4%)	23 (34.8%)	74 (42.5%)	
Unfavorable Intermediate	50 (20.8%)	15 (22.7%)	35 (20.1%)	
High Risk	93 (38.8%)	28 (42.4%)	65 (37.4%)	
Radical prostatectomy approach				0.079
Open	4 (1.7%)	2 (2.9%)	2 (1.2%)	
Laparoscopic	130 (54.6%)	43 (63.2%)	87 (51.2%)	
Robot-assisted	104 (43.7%)	23 (33.8%)	81 (47.6%)	
Time from RP to BCR, median (IQR), months	18.8 (8.3–36.3)	15.5 (9.0–44.5)	20.9 (7.9–34.5)	0.855
PSA at inclusion, median (IQR), ng/mL	0.34 (0.26–0.49)	0.33 (0.27–0.44)	0.34 (0.26–0.51)	0.458
PSA Doubling Time, median (IQR), months	7.0 (4.0–11.0)	7.0 (4.0–13.0)	7.0 (4.0–11.0)	0.416
Risk Group at inclusion				0.952
Intermediate Risk	74 (29.6%)	22 (29.3%)	52 (29.7%)	
High Risk	176 (70.4%)	53 (70.7%)	123 (70.3%)	
Molecular imaging performed	213 (85.9%)	63 (85.1%)	150 (86.2%)	0.824
PET (choline or PSMA)	172 (69.4%)	54 (73.0%)	118 (67.8%)	0.420
MRI	76 (30.6%)	17 (23.0%)	59 (33.9%)	0.087
Conventional imaging	33 (13.3%)	6 (8.1%)	27 (15.5%)	0.116

Data are presented as the number (percentage) of patients with available data unless otherwise indicated. IQR, interquartile range; ECOG, Eastern Cooperative Oncology Group; PSA, Prostate-Specific Antigen; NCCN, National Comprehensive Cancer Network; RP, Radical prostatectomy; BCR, Biochemical Recurrence; PET, Positron Emission Tomography; PSMA, Prostate-Specific Membrane Antigen; MRI, Magnetic Resonance Imaging.

### 4.2 Treatment characteristics

Salvage radiotherapy fractionation schedules varied according to institutional practice. Moderate hypofractionation was delivered in 70% of patients (175/250). The most commonly used regimen was 62.5 Gy in 25 fractions (80.5%), followed by 65.25 Gy in 29 fractions (7.5%), 60 Gy in 24 fractions (4.6%), and 52.5 Gy in 20 fractions (2.9%). Conventional fractionation was delivered in 30% of patients (75/250), typically consisting of 66–70 Gy in 33–35 fractions. The distribution of radiotherapy fractionation schedules is illustrated in Fig. 1B, highlighting the predominance of the 62.5 Gy regimen within the hypofractionated group.

Elective pelvic nodal irradiation was administered in 31.6% of patients overall and was significantly more frequent in the hypofractionation group compared with the conventional fractionation group (39.4% vs 13.3%, *p* = 0.001).

Detailed treatment characteristics are presented in Table 2.

**Table 2:** Radiotherapy and treatment characteristics, including fractionation schedules, pelvic nodal irradiation, and ADT allocation.

	Total N = 250	Conventional fractionation group (n = 75)	Hypofractionation group (n = 175)	p
Radiation therapy treatment				
Type of RT				0.001
VMAT	215 (87.8%)	56 (76.7%)	159 (92.4%)	
IMRT	30 (12.2%)	17 (23.3%)	13 (7.6%)	
Prostate bed dose, median (range), Gy	–	68 (66–70)	62.5 (52.5–65.25)	–
Main schedules delivered (%)	–	66/33f (33.3), 68/34f (22.6), 70/35f (40), others 4	52.5/20f (2.3), 60/24f (4), 62.5/25f (81.7), 65.25/29f (7.4), others 4.6	–
Pelvic nodal irradiation				
Pelvic nodal irradiation	79 (31.6%)	10 (13.3%)	69 (39.4%)	0.001
Pelvic dose, median (range), Gy	50 (44–52.5)	50 (44–52.5)	50 (44–52.5)	–
Main schedules delivered (%)	–	66/33f (33.8), 68/34f (23), 70/35f (40.5)	52.5/20f (2.9), 60/24f (4.6), 62.5/25f (80.5), 65.25/29f (7.5)	
Androgen Deprivation Therapy				
ADT allocation				0.064
24 months of ADT	131 (52.4%)	46 (61.3%)	85 (48.6%)	
6 months of ADT	119 (47.6%)	29 (38.7%)	90 (51.4%)	
ADT type				0.015
Triptorelin	77 (30.8%)	15 (20.0%)	62 (35.4%)	
Leuprorelin	173 (69.2%)	60 (80.0%)	113 (64.6%)	

Data are presented as the number (percentage) of patients with available data unless otherwise indicated. RT, Radiation therapy; VMAT, Volumetric Modulated Arc Therapy; IMRT, Intensity Modulated Radiation Therapy; ADT, Androgen Deprivation Therapy.

### 4.3 Oncologic outcomes

At the time of analysis, the median follow-up was 12.7 months (IQR 6.2–20.2). Early oncologic outcomes were favorable. Biochemical relapse occurred in 3.2% of patients. One metastatic event was observed, and no deaths occurred during the follow-up period.

### 4.4 Physician-reported adverse events

Overall, treatment-related side effects were low. Across the entire cohort, grade ≥ 2 genitourinary (GU) adverse events occurred in 20.4% of patients, while grade ≥ 2 gastrointestinal (GI) adverse events occurred in 8.8%. Grade ≥ 3 adverse events were uncommon, occurring in 2% of patients.

When analyzed according to radiotherapy fractionation schedule, grade ≥ 2 GU side effects occurred in 21.7% of patients treated with hypofractionation compared with 17.3% in those receiving conventional fractionation (*p* = 0.431).

Grade ≥ 2 GI adverse events were observed in 10.8% of patients receiving hypofractionation and in 4.0% of those treated with conventional fractionation (*p* = 0.079). Detailed adverse events outcomes are summarized in Table 3.

**Table 3:** Adverse events (≥ grade 2) according to fractionation schedule.

	Pelvic irradiation (*n* = 79)				Restricted to prostate-bed-only RT (*n* = 171)			
	Conv. (*n* = 10)	Hypo. (*n* = 69)	Absolute Risk Differences (95%CI)	p	Conv. (*n* = 65)	Hypo. (*n* = 106)	Absolute Risk Differences (95%CI)	p
Genitourinary adverse events ≥ grade 2								
Any GU adverse event ≥ grade 2	2 (20.0%)	19 (27.5%)	7.5 (−24.8 to 25.9)	1.000	11 (16.9%)	19 (17.9%)	1.0 (−11.5 to 12.1)	1.000
Hematuria	0 (0.0%)	3 (4.3%)	4.3 (−23.6 to 12.0)	1.000	1 (1.5%)	0 (0.0%)	−1.5 (−8.2 to 2.2)	0.380
Urinary incontinence	2 (20.0%)	12 (17.4%)	−2.6 (−34.4 to 15.2)	1.000	5 (7.7%)	10 (9.4%)	1.7 (−8.3 to 10.0)	0.786
LUTS	1 (10.0%)	7 (10.1%)	0.1 (−30.7 to 12.6)	1.000	2 (3.1%)	7 (6.6%)	3.5 (−4.7 to 10.3)	0.485
Urethral stricture	0 (0.0%)	3 (4.3%)	4.3 (−23.6 to 12.0)	1.000	1 (1.5%)	0 (0.0%)	−1.5 (−8.2 to 2.2)	0.380
Acute Urinary Retention	0 (0.0%)	1 (1.5%)	1.4 (−26.3 to 7.8)	1.000	2 (3.1%)	0 (0.0%)	−3.1 (−10.5 to 1.1)	0.143
Other acute urinary adverse events	1 (10.0%)	13 (18.8%)	8.8 (−22.5 to 22.4)	0.681	8 (12.3%)	11 (10.4%)	−1.9 (−13.0 to 7.4)	0.803
Other chronic urinary adverse events	1 (10.0%)	7 (10.1%)	0.1 (−30.7 to 12.6)	1.000	2 (3.1%)	3 (2.8%)	−0.2 (−7.9 to 5.4)	1.000
Gastrointestinal adverse events ≥ grade 2								
Any GI adverse event ≥ grade 2	0 (0.0%)	12 (17.4%)	17.4 (−11.3 to 28.0)	0.345	3 (4.6%)	7 (6.6%)	2.0 (−6.8 to 9.1)	0.744
Fecal incontinence	0 (0.0%)	1 (1.5%)	1.4 (−26.3 to 7.8)	1.000	2 (3.1%)	0 (0.0%)	−3.1 (−10.5 to 1.1)	0.143
Increased stool frequency	0 (0.0%)	5 (7.2%)	7.2 (−20.8 to 15.9)	1.000	1 (1.5%)	2 (1.9%)	0.3 (−6.5 to 5.2)	1.000
Tenesmus	0 (0.0%)	5 (7.2%)	7.2 (−20.8 to 15.9)	1.000	0 (0.0%)	3 (2.8%)	2.8 (−3.1 to 8.0)	0.289
Rectal bleeding	0 (0.0%)	3 (4.3%)	4.3 (−23.6 to 12.0)	1.000	1 (1.5%)	1 (0.9%)	−0.6 (−7.3 to 3.8)	1.000
Other acute rectal adverse events	0 (0.0%)	7 (10.1%)	10.1 (−18.1 to 19.5)	0.586	1 (1.5%)	5 (4.7%)	3.2 (−4.0 to 9.2)	0.410
Other chronic rectal adverse events	0 (0.0%)	3 (4.3%)	4.3 (−23.6 to 12.0)	1.000	1 (1.5%)	1 (0.9%)	−0.6 (−7.3 to 3.8)	1.000
Severe adverse events								
Grade ≥ 3 events (any)	0 (0.0%)	4 (5.8%)	5.8 (−22.2 to 14.0)	1.000	1 (1.5%)	0 (0.0%)	−1.5 (−8.2 to 2.2)	0.380

Absolute risk difference defined as Hypo minus Conv; 95% confidence intervals were calculated using the Newcombe–Wilson method. Conv., conventional fractionation (66–70 Gy/33–35f); Hypo., moderate hypofractionation (52.5–65.25 Gy/20–29f); LUTS, lower urinary tract symptoms. *Statistically significant (*p* < 0.05). GU, Genitourinary; GI, Gastrointestinal.

Despite the higher use of pelvic nodal irradiation in the hypofractionation group, no statistically significant differences in GU or GI side effects were observed between fractionation schedules. On multivariable analysis(Table 4.), increasing age was independently associated with a higher risk of grade ≥ 2 GU adverse events (OR 1.07; 95% CI 1.01–1.12; *p* = 0.009), while pelvic nodal irradiation was associated with an increased risk of grade ≥ 2 GI side effects (OR 3.05; 95% CI 1.23–7.59; *p* = 0.017).

**Table 4:** Univariate and multivariate analysis of factors associated with grade ≥ 2 GU and GI adverse events.

	Univariate logistic regression	Multivariate logistic regression⁎
GU or GI adverse event ≥ grade 2	OR [95%CI], p	OR [95%CI], p
Age, years	1.07 [1.02–1.11], 0.004	1.07 [1.02–1.12], 0.002
PSA at diagnosis, ng/mL	0.224	
Gleason score	0.183	
Pathologic T stage	0.376	
Positive margins	0.591	
Lymph node involvement	0.492	
NCCN Risk Group at diagnosis	0.334	
Radical prostatectomy approach	0.450	
Time from RP to BCR, months	0.760	
PSA at inclusion, ng/mL	0.156	
PSA Doubling Time, months	0.811	
NCCN Risk Group at inclusion	0.764	
Radiotherapy fractionation	0.186	
Prostate bed dose, Gy	0.424	
Pelvic nodal irradiation	2.06 [1.14–3.71], 0.016	2.36 [1.28–4.35], 0.006
Genitourinary adverse event ≥ grade 2	OR [95%CI], p	OR [95%CI], p
Age, years	1.07 [1.01–1.12], 0.009	1.07 [1.01–1.12], 0.009
PSA at diagnosis, ng/mL	0.097	–
Gleason score	0.388	
Pathologic T stage	0.137	
Positive margins	0.622	
Lymph node involvement	0.956	
NCCN Risk Group at diagnosis	0.653	
Radical prostatectomy approach	0.572	
Time from RP to BCR, months	0.281	
PSA at inclusion, ng/mL	0.063	–
PSA Doubling Time, months	0.488	
NCCN Risk Group at inclusion	0.471	
Radiotherapy fractionation	0.432	
Prostate bed dose, Gy	0.621	
Pelvic nodal irradiation	0.101	–
Gastrointestinal adverse event ≥ grade 2	OR [95%CI], p	OR [95%CI], p
Age, years	0.215	
PSA at diagnosis, ng/mL	0.286	
Gleason score	0.709	
Pathologic T stage	0.985	
Positive margins	0.058	
Lymph node involvement	0.162	
NCCN Risk Group at diagnosis	0.268	
Radical prostatectomy approach	0.060	–
Time from RP to BCR, months	0.447	
PSA at inclusion, ng/mL	0.724	
PSA Doubling Time, months	0.325	
NCCN Risk Group at inclusion	0.468	
Radiotherapy fractionation	0.092	–
Prostate bed dose, Gy	0.283	
Pelvic nodal irradiation	2.88 [1.19–7.00], 0.019	3.05 [1.23–7.59], 0.017

⁎ Adjusted by baseline factors with *p* < 0.10.

ADT-related adverse events were infrequent, with grade ≥ 3 events reported in 1.6% of patients.

### 4.5 Patient-reported outcomes

**Fig. 2:**
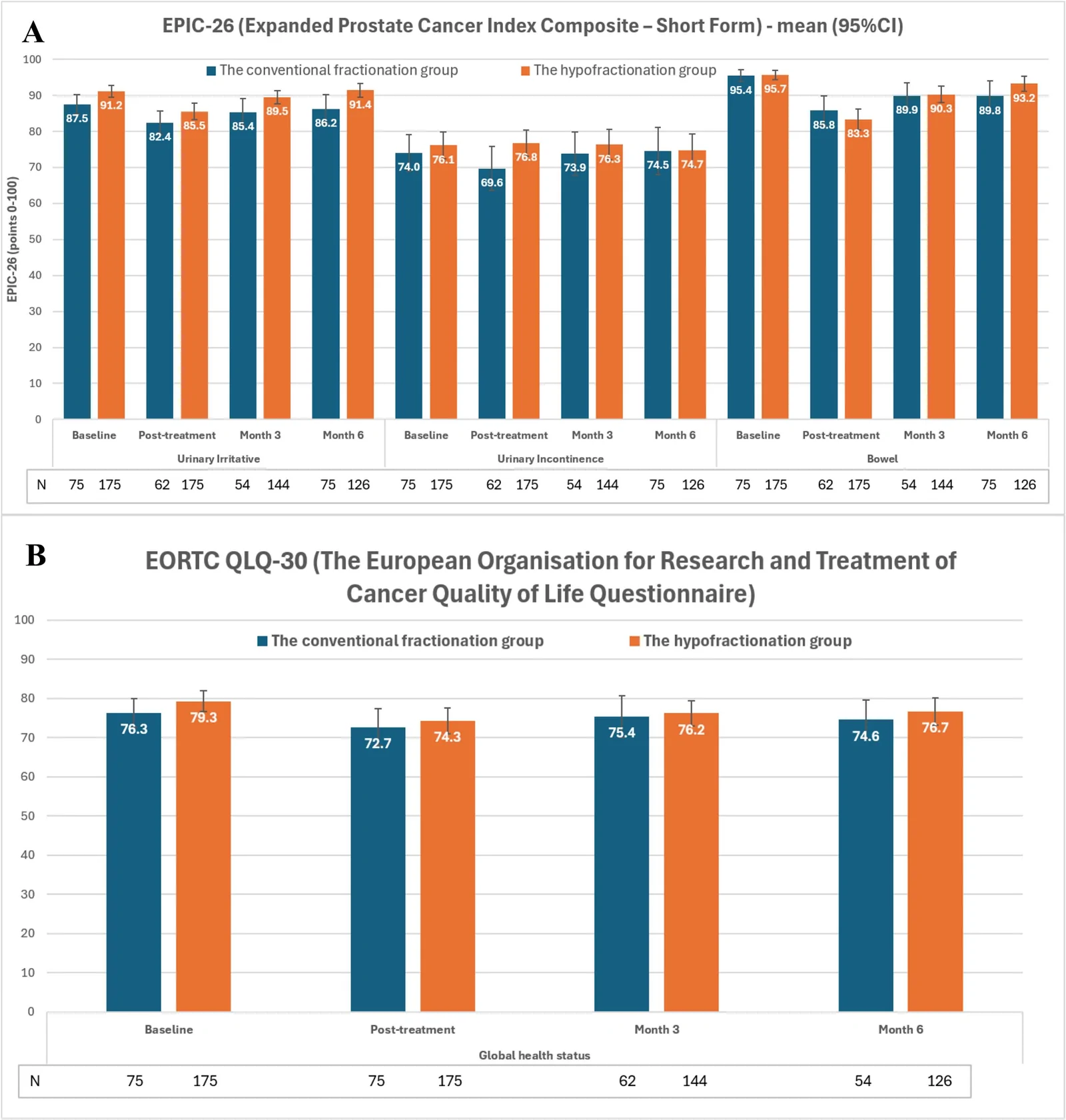
Longitudinal patient-reported outcomes according to radiotherapy fractionation (A. EPIC urinary and bowel domains and B. EORTC QLQ-C30 global health score).

Patient-reported outcomes were available for the majority of patients during follow-up. Completion rates were 81.7% at 3 months and 72.1% at 6 months. Across the evaluated time points, no clinically meaningful differences were observed between hypofractionated and conventionally fractionated radiotherapy schedules in urinary, bowel, or global quality-of-life domains as assessed by the EPIC-26 and EORTC QLQ-C30 questionnaires. Longitudinal patient-reported outcomes are illustrated in Fig. 2.

## 5 Discussion

Moderate hypofractionation has been widely adopted in definitive prostate radiotherapy following multiple randomized trials demonstrating non-inferior oncological outcomes and comparable adverse events compared with conventional fractionation [5], [6], [7]. However, its implementation in the postoperative setting has historically been more cautious due to the limited availability of prospective data and concerns regarding late adverse events in a population with potentially long-life expectancy [13], [14].

In this prospective multicenter analysis of the URONCOR 06–24 trial, early side effects after salvage radiotherapy were low and comparable between moderately hypofractionated and conventionally fractionated schedules. Importantly, no clinically meaningful differences were observed in patient-reported urinary, bowel, or global quality-of-life domains. These findings provide additional prospective evidence supporting the short-term safety and feasibility of moderate hypofractionation in the post-prostatectomy setting [11], [12], [13].

Our results are consistent with emerging randomized data, and this data are summarized in Table 5. The phase III NRG-GU003 trial demonstrated non-inferiority of hypofractionated postoperative radiotherapy (62.5 Gy in 25 fractions) compared with conventional fractionation in terms of patient-reported genitourinary and gastrointestinal adverse events at two years [11]. Similarly, analyses from the RADICALS program comparing 52.5 Gy in 20 fractions with conventional schedules reported low rates of severe side effects and no clinically meaningful differences in patient-reported outcomes [12] Additional prospective series have further supported the feasibility of hypofractionated postoperative radiotherapy, although with relatively limited follow-up [13], [14].

**Table 5:** Adverse events reported in the main prospective studies on hypofractionation in salvage radiotherapy.

AUTHOR	FRACTIONATION	WORST TOXICITY ≥ 3	N	FOLLOW-UP	STUDY
Fersino S 2017 [24]	66 Gy/20 Fx	1.6%	125	18 months	II
Macchia G 2017 [25]	62.5 Gy/25 Fx	4%	124	60 months	II
Viani GA 2022 [26]	52.5 Gy/20 Fx	0%	412	31 months	P
Dubinsky P 2023^[33]^	52.8 Gy/16 Fx	2%	100	61 months	P
Petersen PM 2023 [12]	52.5 Gy/20 Fx	9%	217	58 months	III
Buyyousnouski KM 2024 [11]	66.6 Gy/37 Fx 62.5 Gy/25 Fx	17% 14%	144	24 months	III
Hervás A in press ^[36]^	62.5 Gy/25 Fx	3.8%	389	35 months	II
Couñago F current trial	62.5 Gy/25 Fx 65.25Gy/29Fx 60Gy/24Fx 52.5 Gy/20 Fx	2%	250	12 months	III

N: number of patients; P: prospective; II: trial phase II; III: trial phase III; Gy: Gray; Fx: Fraction.

However, long-term safety remains a critical consideration. A recent study with extended follow-up reported outcomes after dose-escalated hypofractionated post-prostatectomy radiotherapy with a median follow-up of 13.5 years [19]. In that cohort, more than one quarter of patients experienced late grade ≥ 3 adverse events, predominantly genitourinary, including severe complications such as cystectomy and treatment-related mortality. Importantly, only a small proportion of these severe events occurred within the first two years, highlighting that early adverse events assessments may substantially underestimate the long-term risk associated with hypofractionated postoperative radiotherapy [19]. These findings underscore the need for caution and prolonged follow-up when adopting intensified hypofractionated regimens in clinical practice.

An important strength of the present study is that it reflects contemporary clinical practice. Moderate hypofractionation was predominantly delivered using 62.5 Gy in 25 fractions, consistent with emerging standards, while other regimens were used less frequently. In addition, elective pelvic nodal irradiation was delivered in approximately one third of patients and was more frequently used in the hypofractionation group. Despite this imbalance, adverse events outcomes remained comparable between fractionation schedules. Notably, multivariable analysis identified age as an independent predictor of genitourinary adverse events and pelvic nodal irradiation as a predictor of gastrointestinal side effects, whereas fractionation schedule itself was not associated with an increased risk of adverse events. These findings suggest that patient- and treatment-related factors may have a greater impact on early side effects than fractionation per se, consistent with prior observations in postoperative radiotherapy series [[12], [27]].

The association between pelvic nodal irradiation and increased gastrointestinal adverse events is consistent with randomized evidence. In the NRG/RTOG 0534 (SPPORT) trial, the addition of pelvic nodal radiotherapy to prostate bed salvage radiotherapy improved progression-free survival but was associated with higher rates of acute gastrointestinal side effects [27]. Our findings confirm this relationship in a contemporary cohort treated with modern radiotherapy techniques.

Another relevant aspect of the present study is the integration of systemic therapy with salvage radiotherapy. The addition of androgen deprivation therapy (ADT) to postoperative radiotherapy has been associated with improved oncological outcomes in randomized trials and meta-analyses [15], [16]. However, emerging evidence suggests that the benefit of ADT is heterogeneous across different patient populations. In particular, the POSEIDON meta-analysis [28] indicates that selected patients with favorable disease characteristics may derive limited benefit from ADT, supporting a more individualized approach to treatment intensification. Importantly, the present study includes exclusively patients with intermediate- and high-risk biochemical recurrence, a population more likely to benefit from combined modality treatment. In this context, the administration of ADT to all patients in the URONCOR 06–24 trial reflects current clinical practice and is consistent with existing evidence supporting treatment intensification in higher-risk disease. Moreover, the evaluation of ADT duration within this population remains highly clinically relevant, particularly in light of ongoing efforts to optimize the balance between efficacy and adverse events. Together with the widespread use of molecular imaging, particularly PSMA PET/CT [26], these findings illustrate the evolving paradigm toward risk-adapted and personalized management of biochemical recurrence after prostatectomy.

The prospective collection of patient-reported outcomes represents an additional strength. Functional outcomes are particularly relevant in this setting, where urinary and bowel symptoms may significantly impact quality of life. In our cohort, compliance with PRO assessments was high, and no clinically meaningful differences were observed between fractionation schedules across evaluated domains. These findings are consistent with randomized trials incorporating PROs as key endpoints [11], [12].

Several limitations should be acknowledged. First, this analysis was not part of the original trial design, which was primarily conceived to evaluate the duration of androgen deprivation therapy, and the fractionation schedules were not initially intended as a comparative endpoint; moreover, given the heterogeneity in treatment subdivisions during the study period, the present analysis should be considered exploratory. Importantly, this study focused on early adverse events, and longer follow-up will be required to adequately characterize late genitourinary and gastrointestinal toxicity, which may occur several years after postoperative radiotherapy, as demonstrated by long-term data on hypofractionated postoperative radiotherapy [19]. Second, the interaction between ADT and radiotherapy-related adverse events may have been overestimated or underestimated, as treatment allocation was not stratified or randomized according to ADT use. Third, fractionation schedules were not randomized but selected according to institutional practice, potentially introducing selection bias; in particular, the 62.5 Gy regimen was used more frequently in this cohort, which may limit the generalizability of our findings to institutions using different postoperative fractionation schedules. Fourth, there was a substantial imbalance in the use of pelvic nodal irradiation, which may have influenced gastrointestinal toxicity. Although multivariable analysis was performed to mitigate the potential confounding effect of this factor, studies with more balanced treatment cohorts are needed to better determine its independent contribution to adverse events. Nevertheless, the pragmatic nature of treatment allocation reflects real-world clinical practice and may enhance the external validity of the findings

## 6 Conclusion

In conclusion, these exploratory interim data demonstrate acceptable early toxicity with moderately hypofractionated salvage radiotherapy within a contemporary prospective cohort. However, given the non-randomized nature of the fractionation regimens, longer follow-up and robust adjustment for treatment-selection biases are essential before drawing definitive conclusions regarding comparative safety.

## CRediT authorship contribution statement

**Felipe Couñago:** Methodology, Investigation, Formal analysis, Conceptualization. **Carmen González:** Writing – review & editing, Writing – original draft, Validation, Supervision, Methodology, Investigation, Formal analysis, Data curation, Conceptualization. **Alfonso Gómez-Iturriaga:** Writing – review & editing, Writing – original draft, Visualization, Validation, Supervision, Methodology, Investigation, Formal analysis, Data curation, Conceptualization. **Ana Álvarez:** Methodology, Investigation, Formal analysis, Data curation, Conceptualization. **Marina Santos:** Methodology, Investigation, Data curation, Conceptualization. **Ana Boladeras:** Methodology, Investigation, Formal analysis, Data curation, Conceptualization. **Nagore García:** Methodology, Investigation, Formal analysis, Data curation, Conceptualization. **Sandra Fernández:** Methodology, Investigation, Data curation, Conceptualization. **Sandra Guardado:** Methodology, Investigation, Formal analysis, Conceptualization. **Abrahams Ocanto:** Writing – review & editing, Writing – original draft, Methodology, Investigation, Formal analysis, Data curation, Conceptualization. **Daniela Gonsalves:** Methodology, Investigation, Formal analysis, Data curation, Conceptualization. **Luis Glaría:** Methodology, Investigation, Formal analysis, Data curation, Conceptualization. **Ana Castaño:** Methodology, Investigation, Formal analysis, Data curation, Conceptualization. **Patricia Martín:** Methodology, Investigation, Formal analysis, Data curation, Conceptualization. **Laura Montezuma:** Methodology, Investigation, Formal analysis, Data curation, Conceptualization. **Arantxa Mera:** Methodology, Investigation, Formal analysis, Data curation, Conceptualization. **Joel Mases:** Methodology, Investigation, Formal analysis, Data curation, Conceptualization. **Gerard Meca:** Methodology, Investigation, Formal analysis, Data curation, Conceptualization. **Víctor Duque-Santana:** Methodology, Investigation, Formal analysis, Data curation, Conceptualization. **Jesús Olivera:** Methodology, Investigation, Formal analysis, Data curation, Conceptualization. **Sara Pérez:** Methodology, Investigation, Formal analysis, Data curation, Conceptualization. **Ivan Henriquez:** Methodology, Investigation, Data curation, Conceptualization. **Bárbara Malavé:** Methodolgy, Investigation, Data curation, Conceptualization. **Aurora Rodríguez:** Methodology, Investigation, Formal analysis, Data curation, Conceptualization. **Rafael García:** Methodology, Investigation, Formal analysis, Data curation, Conceptualization. **David Büchser:** Methodology, Investigation, Formal analysis, Data curation, Conceptualization. **Josep Garre:** Methodology, Investigation, Formal analysis, Data curation, Conceptualization. **Manuel Altabas:** Methodology, Investigation, Formal analysis, Data curation, Conceptualization. **Jeannette Valero:** Methodology, Investigation, Formal analysis, Data curation, Conceptualization. **Raquel García-Gómez:** Methodology, Investigation, Formal analysis, Data curation, Conceptualization. **Miguel Berenguer:** Methodology, Investigation, Formal analysis, Data curation, Conceptualization. **Ángel Calvo:** Methodology, Investigation, Formal analysis, Data curation, Conceptualization. **Rafael Ordoñez:** Methodology, Investigation, Formal analysis, Data curation, Conceptualization. **Patricia Willisch:** Methodology, Investigation, Formal analysis, Data curation, Conceptualization. **Aldara Candal:** Methodology, Investigation, Formal analysis, Data curation, Conceptualization. **Neus Aymar:** Methodology, Investigation, Formal analysis, Data curation, Conceptualization. **Esther Mayrata:** Methodology, Investigation, Formal analysis, Data curation, Conceptualization. **Fernando López-Campos:** Writing – review & editing, Writing – original draft, Visualization, Validation, Supervision, Methodology, Investigation, Formal analysis, Data curation, Conceptualization.

## Declaration of competing interest

The authors declare the following financial interests/personal relationships which may be considered as potential competing interests: Felipe Counago reports statistical analysis was provided by Spanish Society of Radiation Oncology. Felipe Counago reports a relationship with Janssen Pharmaceuticals Inc. that includes: board membership, speaking and lecture fees, and travel reimbursement. Felipe Counago reports a relationship with Recordati Industria Chimica e Farmaceutica SpA that includes: consulting or advisory and travel reimbursement. Felipe Counago reports a relationship with AstraZeneca Pharmaceuticals LP that includes: consulting or advisory and travel reimbursement. Felipe Counago reports a relationship with Astellas Pharma US Inc. that includes: consulting or advisory and travel reimbursement. Felipe Counago reports a relationship with Bayer Corporation that includes: speaking and lecture fees. Sandra Fernandez reports a relationship with Janssen Pharmaceuticals Inc. that includes: consulting or advisory, speaking and lecture fees, and travel reimbursement. Sandra Fernandez reports a relationship with Astellas Pharma US Inc. that includes: consulting or advisory and speaking and lecture fees. Sandra Fernandez reports a relationship with Bayer Corporation that includes: consulting or advisory. Sandra Fernandez reports a relationship with Recordati Industria Chimica e Farmaceutica SpA that includes: consulting or advisory, speaking and lecture fees, and travel reimbursement. Fernando Lopez-Campos reports a relationship with Janssen Pharmaceuticals Inc. that includes: consulting or advisory, speaking and lecture fees, and travel reimbursement. Fernando Lopez-Campos reports a relationship with Astellas Pharma US Inc. that includes: consulting or advisory, speaking and lecture fees, and travel reimbursement. Fernando Lopez-Campos reports a relationship with Bayer Corporation that includes: consulting or advisory, speaking and lecture fees, and travel reimbursement. Fernando Lopez-Campos reports a relationship with Recordati Industria Chimica e Farmaceutica SpA that includes: consulting or advisory, speaking and lecture fees, and travel reimbursement. Fernando Lopez-Campos reports a relationship with Ipsen that includes: consulting or advisory, speaking and lecture fees, and travel reimbursement. Felipe Counago reports a relationship with Ipsen that includes: consulting or advisory and travel reimbursement. Sandra Guardado reports a relationship with Ipsen that includes: speaking and lecture fees. Sandra Guardado reports a relationship with Janssen Pharmaceuticals Inc. that includes: consulting or advisory and speaking and lecture fees. Sandra Guardado reports a relationship with Recordati Industria Chimica e Farmaceutica SpA that includes: consulting or advisory and speaking and lecture fees. Sandra Guardado reports a relationship with Bayer Corporation that includes: travel reimbursement. Alfonso Gomez-Iturriaga reports a relationship with Bayer Corporation that includes: consulting or advisory and speaking and lecture fees. Alfonso Gomez-Iturriaga reports a relationship with Astellas Pharma Inc. that includes: consulting or advisory and speaking and lecture fees. Alfonso Gomez-Iturriaga reports a relationship with Recordati Industria Chimica e Farmaceutica SpA that includes: consulting or advisory and speaking and lecture fees. Alfonso Gomez-Iturriaga reports a relationship with Accord Healthcare Inc. that includes: consulting or advisory and speaking and lecture fees. Alfonso Gomez-Iturriaga reports a relationship with Bayer Corporation that includes: consulting or advisory. Alfonso Gomez-Iturriaga reports a relationship with Elekta that includes: consulting or advisory. If there are other authors, they declare that they have no known competing financial interests or personal relationships that could have appeared to influence the work reported in this paper.
